# Sociodemographic correlates of cervix, breast and oral cancer screening among Indian women

**DOI:** 10.1371/journal.pone.0265881

**Published:** 2022-05-11

**Authors:** Zhu Changkun, Ghose Bishwajit, Lu Ji, Shangfeng Tang

**Affiliations:** 1 Women’s Hospital, School of Medicine Zhejiang University, Hangzhou, China; 2 School of Medicine and Health Management, Tongji Medical College, Huazhong University of Science and Technology, Wuhan, Hubei, China; Charite Universitatsmedizin Berlin, GERMANY

## Abstract

**Introduction:**

Cervix, breast and oral cancers account for about one-third of all cancers in India which as a group is a major contributor to all non-communicable disease-related morbidity and mortality among women. Existing evidence suggests that early diagnosis plays a pivotal role in the prevention and intervention of these cancers, and many community-based early screening and awareness programs have been in place in developed countries. Currently, there is not enough research evidence regarding the sociodemographic correlates of cervix, breast and oral cancer screening among Indian women. In the present study, we aimed to assess the self-reported percentage and sociodemographic factors associated with the use of these three types of cancer screening services among Indian women aged 15–49 years.

**Methods:**

Data were collected from National Family Health Survey conducted during 2015–16. Sample population was 699,686 women aged 15–49 years. Associations between self-reported cervical, breast and oral cancer screening status and the associated sociodemographic factors were analyzed using multivariable logistic regression methods.

**Results:**

The percentage of screening for cervical (21%), breast (8.95%), and oral cancers (13.45%) varied significantly across the population sub-groups. Higher age, urban residence, higher education, having employment, health insurance, use of electronic media, higher household wealth quintile, having healthcare autonomy, showed a positive effect on taking screening services. Further analyses revealed that the strength of the associations varied considerably between urban and rural residents, denoting the need for region-specific intervention strategies. Sex of household head, age, watching TV, using radio, and having health insurance were the most significant contributors to the outcome effects.

**Conclusions:**

The present study provides important insights regarding the current scenario of seeking cancer screening services among women in India. These findings could inform policy analysis and make an avenue for further in-depth analysis for future studies. Our findings conclude that cancer prevention policies should focus on leveraging the positive effects of better socioeconomic status, employment, health insurance ownership, exposure to electronic media, and better healthcare autonomy to improve the cancer screening service uptake among Indian women.

## Background

The objective of the present study is to assess the prevalence of sociodemographic predictors of using cervical, breast, and oral cancer screening among Indian women aged 15–49 years. The prevalence of oral [1] and cervical cancer (Kerala) [2] screening behaviour was previously studied at the sub-national level. However, the current evidence base is shockingly scarce given the fact that the country has some of the highest cancer rates in the world [3], which is projected to double in the next two decades [4]. Although the prevalence is lower relative to the high-income nations, current statistics suggest that the situation is alarming due to the relatively younger age structure of the population. According to a local report, only four types (breast, cervical, oral, and lung) contribute to over two-fifths (41%) of the national cancer burden, with breast and cervical cancer being the most prevalent among women [5] and oral cancer among men [6]. Despite this situation, no nationwide program has been put in place to provide basic preventive measures such as screening services [7].

Screening for breast and oral cancers are highly rewarding in terms of reducing mortality rates. Various screening methods are currently available which are generally provided through primary care centres [8–10], and the use of these screening the advanced economies is quite common, unlike in India where the practice is rare [7]. Unfortunately, cancer prevention programs in India do not get enough attention despite the rising burden of the disease, its high social and economic costs, and the availability of cost-effective screening methods. Therefore, identifying the sub-population deprived of the potentially life-saving diagnostic services can provide vital information for healthcare programmers and researchers fighting against the growing cancer burden in the country.

Although breast and cervical cancers are currently the largest contributors to the total cancer burden among Indian women, they are generally more manageable and have better survival rates than oral cavity cancer (OCC) [11]. OCC ranks third in terms of prevalence and fifth in terms of overall cancer mortality in India [12]. The high rate of OCC is attributed to a large extent to the widespread consumption of tobacco products, especially the smokeless types (SLT) such as mishri, pan tobacco, and gutkha [13], which unfortunately have greater cultural acceptance among women [14]. According to the Global Adult Tobacco Survey (GATS), the prevalence of SLT consumption among Indian women was remarkably higher (17%) compared with that for smoking tobacco (2%) [14]. It was reported that SLT consumption is responsible for 52% of all oral cancer cases [15]. Given the well-established relationship between smoking of any type of cancer [16], especially cervical [17, 18], breast [19–21] and oral cancers [16, 22–24], the risk expansion of these diseases remains high among Indian women.

## Methods

### Survey and data source

Data for this study was collected from round 4 of the National Family Health Survey (NFHS) of India. This is a part of a Demographic and Health Survey program that conducts population surveys in low-middle income countries on nationally representative samples. In India, the NFHS is one of the largest surveys of the kind that collects information from various demographic, socioeconomic and health indicators among adult men, women and under-five children. NFHS was conducted by the Ministry of Health and Family Welfare and the fieldwork lasted from January 2015 to December 2016. The design consisted of a multistage stratified sampling method that included households in both urban and rural areas, and data collection was facilitated by Computer Assisted Personal Interviewing (CAPI). In total, 699 686 women were interviewed in the survey for a response rate of 97 percent. These details are available from the final report of the NFHS survey (http://rchiips.org/nfhs/NFHS-4Reports/India.pdf).

### Description of outcome and explanatory variables

The outcome variables included self-reported status of cervix, breast and oral cavity cancer. All these variables were recorded in binary format: ‘Yes’ and ‘No.’

A literature review was conducted in PubMed to identify original studies on cancer screening behaviour among women. The aim was to facilitate the selection of relevant socioeconomic factors for the analysis. Based on the review, the following variables were identified and extracted from the dataset (Table 1): [10, 25–28].

**Table 1:** Description of the study variables.

Variables	Categories
Age groups	15-19/ 20-24/ 25-29/ 30-34/ 35-39/ 40-44/ 45–49
Education	No Education/ Primary/ Secondary/ Higher
Occupation	None/ White Collar/ Blue Collar
Has insurance	No/ Yes
Radio use	Not At All/ Less Than Once per Week/ At Least Once per Week/ Almost Every Day
TV use	Not At All/ Less Than Once per Week/ At Least Once per Week/ Almost Every Day
Religion	Hindu/ Muslim/ Other
Wealth quintile	Poorest/ Poorer/ Middle/ Richer/ Richest);
Household head’s sex	Male/ Female
Healthcare decision-maker	Respondent alone/ Respondent & Husband together/ Husband alone);
Residence	Urban/ Rural
Husband’s education	No Education/ Primary/ Secondary/ Higher

### Data analysis

Stata 16 was used for all statistical analyses. Participants who did not have data on cancer screening were excluded from the analysis. As the survey used a multistage cluster sampling method, we used the *svy* command in Stata to adjust for sampling weight, strata and primary sampling units. The prevalence of using cervical, breast and oral cancers were presented as percentages across the explanatory variables. Following the descriptive analysis, bivariable and multivariable regression models were carried out without taking the categories into account. These results were presented as forest plots containing odds ratios and 95% confidence intervals. In the next step, the analysis was repeated for multivariable regression models by showing the odds ratios for all categories for a better understanding of the inter-category variations. At the last step, two more multivariable analyses were conducted for each of the outcome variables stratified by urban and rural samples. The decision to conduct the stratified was guided by previous studies that demonstrated significant urban-rural differences in healthcare screening behaviour [29, 30]. For all analyses, a value of p < 0.05 was considered statistically significant.

### Ethics statement

Not applicable. Specific ethics approval was not required for this study since it was a secondary publicly available dataset. Details were available at the website: http://rchiips.org/nfhs/NFHS-4Reports/India

## Results

The prevalence of breast cancer, cervix and oral cavity test was presented in Table 2. The prevalence of cervix screening (21.06%) was the highest among the three, followed by having an oral cavity (13.45%) and breast screening (8.98%). About one-fifth (18.88%) of the w omen had at least one test and 3.3% had all three screening tests taken (not shown in the table). A higher percentage of women who took these tests were aged between 25 to 39 years, had secondary level education, had no employment and had health insurance, used TV almost every day, were followers of Hinduism, lived in households with higher wealth quintiles and were headed by men, made healthcare decisions jointly with husband, lived in rural areas, and had husbands with a secondary education.

**Table 2:** Prevalence of breast cancer, cervix and oral cavity test.

	N = 699,686	*Cervix screening*		*Breast screening*		*Oral cavity screening*	
		No (78.94%)	Yes (21.06%)	No (91.02%)	Yes (8.98%)	No (86.55%)	Yes (13.45%)
**Age**							
15–19	124,878	21.5	3.0	18.8	4.0	18.4	10.2
20–24	122,955	18.9	13.0	18.1	13.1	18.1	14.1
25–29	115,076	15.7	19.0	16.2	18.7	16.4	16.7
30–34	97,048	12.6	18.0	13.4	17.4	13.6	15.4
35–39	90,433	11.8	17.2	12.5	17.2	12.6	15.8
40–44	76,627	10.0	15.2	10.7	15.0	10.7	14.1
45–49	72,669	9.5	14.7	10.2	14.5	10.2	13.8
**Education**							
No Education	196,556	27.4	27.8	27.8	24.3	28.3	21.4
Primary	88,29	12.2	13.4	12.4	13.0	12.7	11.1
Secondary	334,927	47.5	46.6	47.2	48.5	47.0	49.5
Higher	79,913	12.9	12.2	12.6	14.2	12.0	18.1
**Occupation**							
None	85,138	70.6	66.9	70.0	67.6	69.9	68.7
White Collar	14,994	12.4	13.0	12.5	13.5	12.2	14.8
Blue Collar	22,219	17.0	20.1	17.6	19.0	17.9	16.5
**Has insurance**							
No	574,718	80.8	75.6	80.0	76.4	80.0	76.8
Yes	124,968	19.2	24.4	20.0	23.6	20.0	23.2
**Radio user**							
Not At All	585,631	84.3	84.2	84.6	81.8	84.9	80.1
Less Than Once /Week	41,394	5.2	4.9	5.2	5.3	5.1	6.0
At Least Once /Week	43,668	6.2	6.2	6.1	7.0	6.1	7.3
Almost Every Day	28,993	4.2	4.7	4.2	5.9	4.0	6.6
**TV user**							
Not At All	170,542	24.7	15.9	23.7	14.6	24.1	13.4
Less Than Once/ Week	52,768	6.4	5.3	6.3	4.8	6.4	4.8
At Least Once/ Week	81,777	10.1	9.1	10.0	9.3	10.0	9.0
Almost Every Day	394,599	58.7	69.7	60.1	71.3	59.5	72.8
**Religion**							
Hindu	519,281	80.6	80.4	80.7	79.1	81.2	76.2
Muslim	94,591	14.4	11.7	14.0	12.1	13.8	13.9
Other	85,814	15.0	7.9	5.3	8.8	5.0	9.9
**Wealth quintile**							
Poorest	133,249	19.8	10.6	18.6	9.6	19.1	8.3
Poorer	149,466	20.8	15.4	20.1	14.7	20.5	13.0
Middle	147,168	20.4	21.0	20.6	20.6	20.8	18.9
Richer	138,502	20.1	24.9	20.7	25.3	20.5	25.4
Richest	131,301	18.9	28.2	20.0	29.8	19.1	34.5
**Household head’s sex**							
Male	604,912	86.2	87.7	86.5	87.0	86.5	86.6
Female	94,774	13.8	12.3	13.5	13.0	13.5	13.4
**Healthcare decision maker**							
Respondent alone	9,438	12.0	11.9	12.1	11.4	11.9	12.6
Respondent & Husband	56,238	62.2	63.3	62.2	64.6	62.0	65.3
Husband alone	21,135	25.8	24.8	25.7	24.0	26.1	22.1
**Residence**							
Urban	204,735	33.3	39.2	33.9	41.4	33.4	43.4
Rural	494,951	66.7	60.8	66.1	58.6	66.6	56.6
**Husband’s education**							
No Education	18,251	21.3	16.1	20.6	14.2	20.8	14.1
Primary	13,648	15.4	13.6	15.1	13.6	15.3	12.6
Secondary	48,314	49.8	55.0	50.8	55.2	50.7	55.1
Higher	12,093	13.5	15.3	13.6	17.0	13.2	18.3

**Fig 1:**
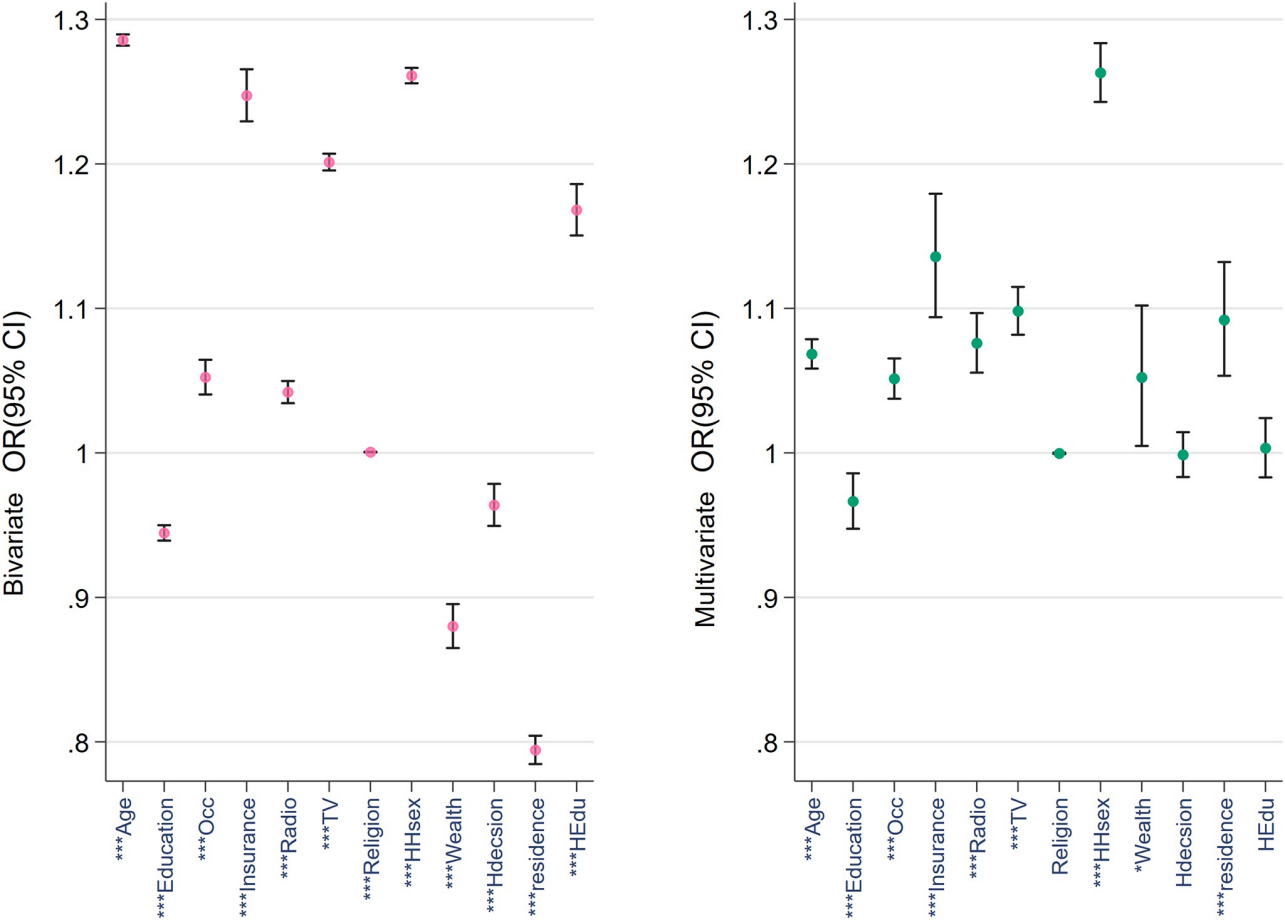
Bivariable and multivariable correlation among cervix test and the covariables. N.B.: * p < 0.05, ** p < 0.01, *** p < 0.001. HHsex = household head’s sex; Hdecision = healthcare decision maker, HEdu = husband’s education.

**Fig 2:**
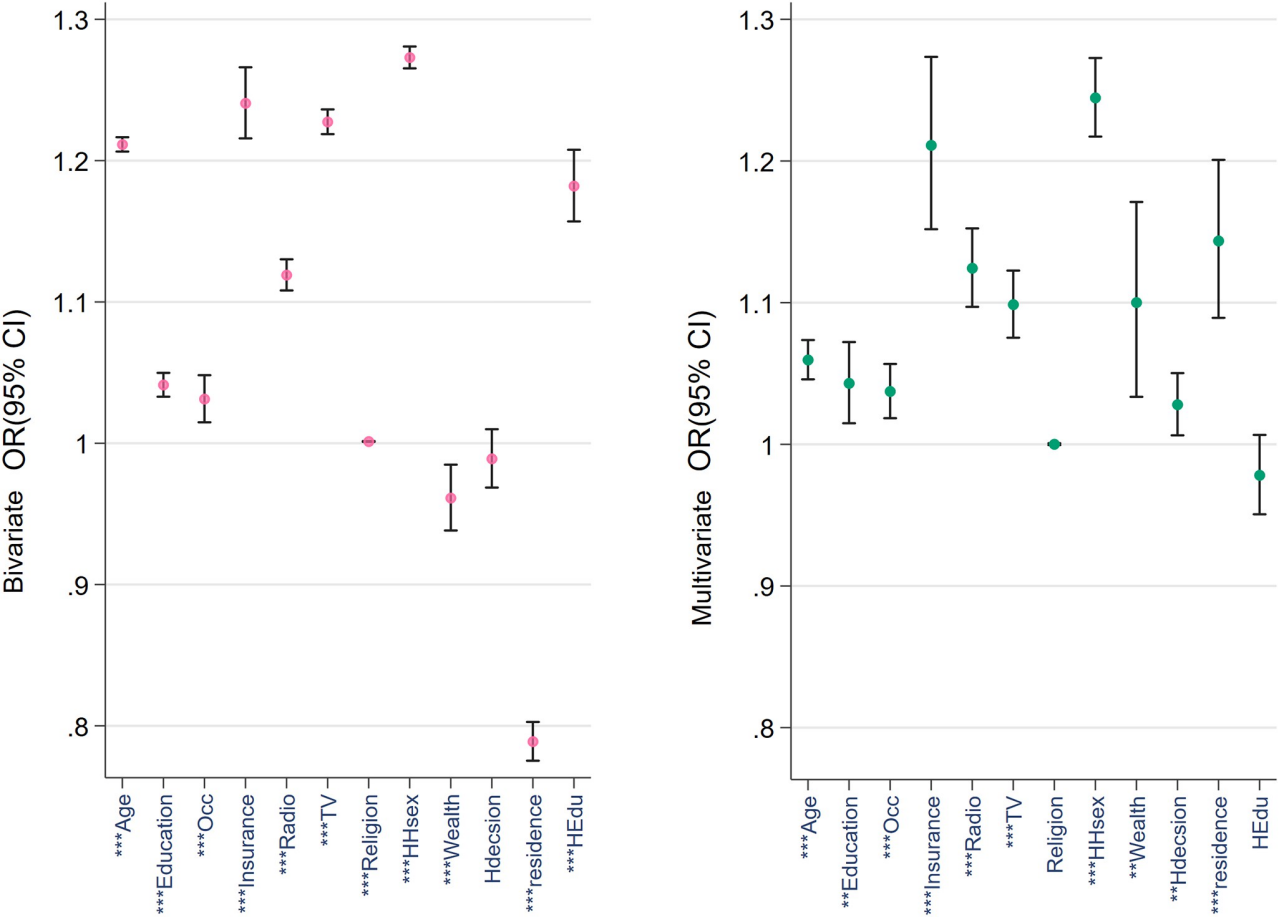
Bivariable and multivariable correlation among breast screening and the covariables. N.B.: * p < 0.05, ** p < 0.01, *** p < 0.001. HHsex = household head’s sex; Hdecision = healthcare decision maker, HEdu = husband’s education.

**Fig 3:**
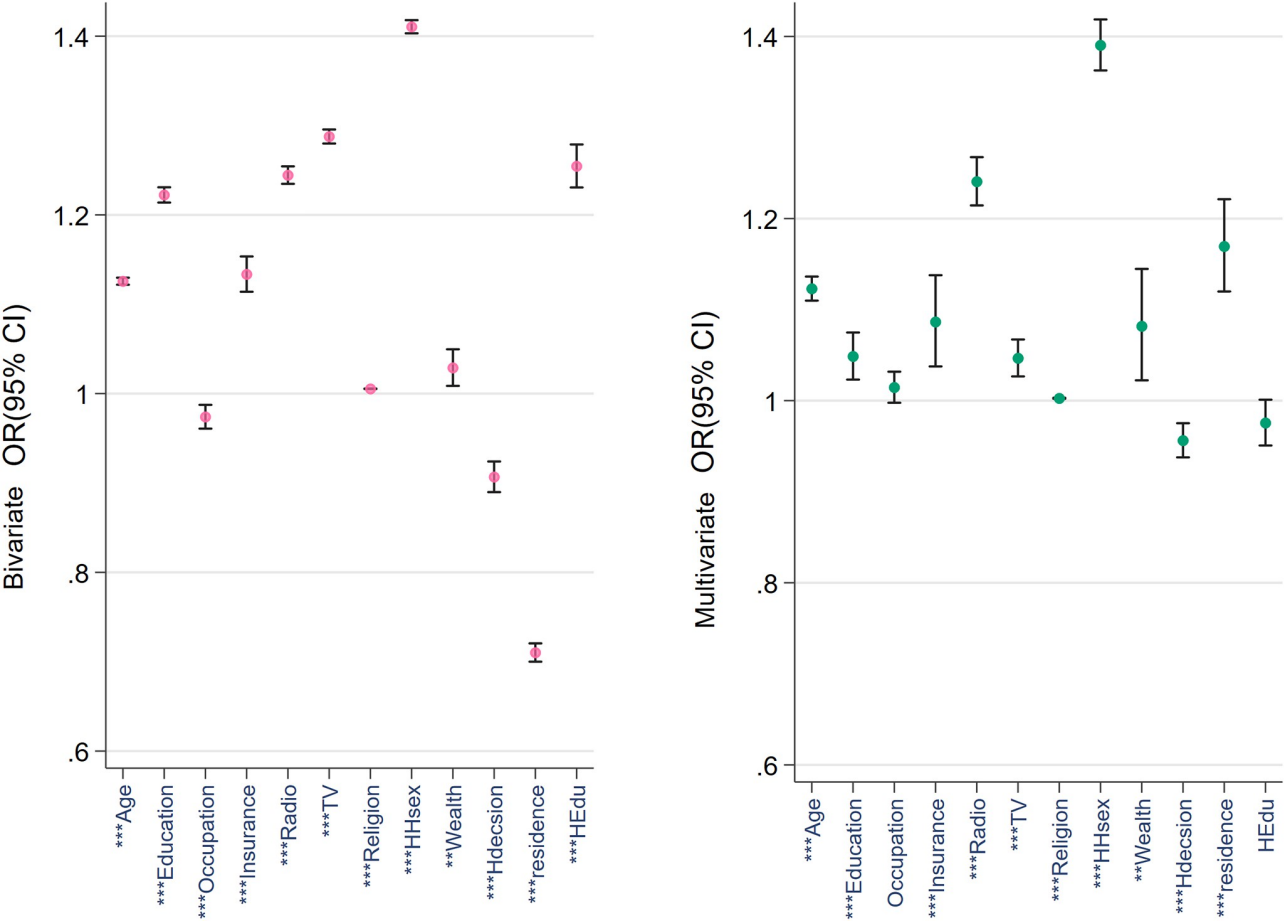
Bivariable and multivariable correlation among breast oral cavity test and the covariables. N.B.: * p < 0.05, ** p < 0.01, *** p < 0.001. HHsex = household head’s sex; Hdecision = healthcare decision maker, HEdu = husband’s education.

Figs 1–3 provide a comparative view of the bivariable and multivariable associations between the three types of cancer screenings and the covariables. Noticeable among all three figures is the strength of the association between age, education, wealth quintile, insurance ownership and household head’s sex. Husband’s education was not found to be associated with any of the outcomes in the multivariable analyses.

The multivariable analyses were further extended to produce a better picture of intercategory variability, both for the overall sample (Table 3) and then for the urban and rural samples separately (Table 4). Findings from Table 3 indicate a positive association between cancer screening (all three) with higher age groups. Women in the highest age group (45–49 years) had the highest odds of having cervix [Odds ratio = 2.44, 95%CI = 2.17,2.74], breast [Odds ratio = 2.08, 95%CI = 1.76,2.45] and oral cavity screening [Odds ratio = 2.49, 95%CI = 2.15,2.90]. Women with higher education had higher odds of breast [Odds ratio = 1.21,1.10,1.33] and oral cavity screening [Odds ratio = 1.29, 95%CI = 1.19,1.40] only but not of cervix screening. Compared with women who had no employment, those who were employed had higher odds of taking all three screenings. Similarly, positive association was observed for health insurance ownership as well, with the odds being 1.17 [95%CI = 1.13,1.22], 1.25 [95%CI = 1.19,1.31], 1.14 [95%CI = 1.09,1.20] times higher for cervix, breast and oral cavity screening respectively. Regular use of radio and television also showed significantly positive associations with the uptake of all three screenings. Households wealth quintile showed strong positive association as well, with the association being strongest for those in the highest wealth quintile: cervix [Odds ratio = 2.56, 95%CI = 2.39,2.75], breast [Odds ratio = 2.33, 95%CI = 2.11,2.57] and oral cavity [Odds ratio = 3.59, 95%CI = 3.27,3.94]. Women who made healthcare decision alone had higher odds of breast [Odds ratio = 1.13, 95%CI = 1.06,1.21] and lower odds of having oral cavity screening [Odds ratio = 0.93, 95%CI = 0.88,0.99]. Urban residents had higher odds higher for cervix [Odds ratio = 1.11, 95%CI = 1.07,1.15], breast [Odds ratio = 1.16, 95%CI = 1.10,1.22] and oral cavity screening [Odds ratio = 1.22, 95%CI = 1.17,1.28].

**Table 3:** Factors associated with cervix, breast and oral cavity screening among Indian women.

	*Cervix screening*	*Breast screening*	*Oral cavity screening*
**Age (15–19 years)**			
20–24	1.82*** [1.63,2.04]	1.56*** [1.32,1.83]	1.34*** [1.15,1.56]
25–29	2.08*** [1.86,2.33]	1.79*** [1.52,2.10]	1.74*** [1.50,2.01]
30–34	2.29*** [2.05,2.56]	2.01*** [1.71,2.36]	2.01*** [1.74,2.33]
35–39	2.33*** [2.09,2.61]	2.05*** [1.74,2.41]	2.35*** [2.03,2.72]
40–44	2.44*** [2.18,2.74]	2.05*** [1.74,2.41]	2.47*** [2.13,2.88]
45–49	2.44*** [2.17,2.74]	2.08*** [1.76,2.45]	2.49*** [2.15,2.90]
**Education (None)**			
Primary	0.98 [0.94,1.03]	1.08* [1.01,1.16]	0.95 [0.89,1.01]
Secondary	0.97 [0.93,1.02]	1.13*** [1.06,1.20]	1.11*** [1.05,1.18]
Higher	0.94 [0.87,1.01]	1.21*** [1.10,1.33]	1.29*** [1.19,1.40]
**Occupation (None)**			
Blue Collar	1.05* [1.01,1.11]	1.01 [0.94,1.08]	1.12*** [1.06,1.19]
White Collar	1.21*** [1.16,1.26]	1.17*** [1.10,1.23]	1.11*** [1.06,1.17]
**Has insurance (No)**			
Yes	1.17*** [1.13,1.22]	1.25*** [1.19,1.31]	1.14*** [1.09,1.20]
**Radio use (Not at all)**			
Less Than Once /Week	1.05 [0.98,1.12]	1.16** [1.06,1.26]	1.36*** [1.26,1.47]
At Least Once /Week	1.08* [1.01,1.15]	1.22*** [1.13,1.33]	1.39*** [1.29,1.49]
Almost Every Day	1.30*** [1.21,1.40]	1.43*** [1.30,1.56]	1.77*** [1.63,1.91]
**TV use (Not at all)**			
Less Than Once/ Week	1.24*** [1.16,1.32]	1.19*** [1.08,1.31]	1.18*** [1.09,1.29]
At Least Once/Week	1.27*** [1.20,1.35]	1.16*** [1.06,1.26]	1.22*** [1.14,1.32]
Almost Every Day	1.36*** [1.29,1.42]	1.34*** [1.25,1.44]	1.25*** [1.17,1.33]
**Religion (Hindu)**			
Muslim	1.26*** [1.20,1.32]	1.25*** [1.18,1.33]	1.83*** [1.74,1.93]
Other	0.99 [0.94,1.04]	1.03 [0.97,1.10]	1.39*** [1.32,1.47]
**Wealth quintile (Poorest)**			
Poorer	1.30*** [1.23,1.38]	1.20*** [1.11,1.31]	1.45*** [1.34,1.57]
Middle	1.61*** [1.52,1.71]	1.57*** [1.44,1.71]	2.08*** [1.92,2.26]
Richer	1.94*** [1.82,2.07]	1.86*** [1.70,2.04]	2.62*** [2.40,2.85]
Richest	2.56*** [2.39,2.75]	2.33*** [2.11,2.57]	3.59*** [3.27,3.94]
**Household head’s sex (Male)**			
Female	1.05* [1.00,1.10]	1.10** [1.03,1.17]	1.06* [1.00,1.12]
**Healthcare decision maker (Respondent)** Alone			
Respondent and Husband/Partner	1.03 [0.98,1.08]	1.13*** [1.06,1.21]	0.93* [0.88,0.99]
Husband/Partner Alone	1.02 [0.97,1.08]	1.16*** [1.07,1.25]	0.86*** [0.81,0.92]
**Residence (rural)**			
Urban	1.11*** [1.07,1.15]	1.16*** [1.10,1.22]	1.22*** [1.17,1.28]
**Husband’s Education (None)**			
Primary	1.04 [0.98,1.10]	0.99 [0.87,1.14]	0.95 [0.89,1.02]
Secondary	1.10 [0.97,1.25]	1.05 [0.98,1.12]	1.02 [0.96,1.09]
Higher	0.96 [0.90,1.03]	0.94 [0.86,1.03]	0.95 [0.87,1.03]

Exponentiated coefficients; 95% confidence intervals in brackets

* *p* < 0.05,

** *p* < 0.01,

*** *p* < 0.001

**Table 4:** Urban-rural differences in the factors associated with cervix, breast and oral cavity screening among Indian women.

	*Cervix screening*		*Breast screening*		*Oral cavity screening*	
	*Urban*	*Rural*	*Urban*	*Rural*	*Urban*	*Rural*
**Age (15–19)**						
20–24	1.40**	1.96***	1.23	1.67***	0.99	1.49***
	[1.11,1.75]	[1.72,2.24]	[0.89,1.71]	[1.38,2.02]	[0.74,1.31]	[1.25,1.78]
25–29	1.46***	2.32***	1.30	1.99***	1.27	1.92***
	[1.17,1.82]	[2.04,2.64]	[0.95,1.79]	[1.65,2.39]	[0.96,1.67]	[1.61,2.29]
30–34	1.67***	2.51***	1.64**	2.13***	1.46**	2.24***
	[1.34,2.08]	[2.21,2.86]	[1.19,2.25]	[1.76,2.56]	[1.11,1.92]	[1.88,2.66]
35–39	1.71***	2.55***	1.69**	2.13***	1.72***	2.58***
	[1.37,2.13]	[2.23,2.90]	[1.23,2.32]	[1.77,2.58]	[1.31,2.26]	[2.16,3.08]
40–44	1.89***	2.59***	1.71**	2.12***	1.89***	2.68***
	[1.51,2.36]	[2.26,2.95]	[1.24,2.35]	[1.75,2.56]	[1.43,2.49]	[2.24,3.21]
45–49	1.99***	2.51***	1.84***	2.07***	1.91***	2.61***
	[1.59,2.49]	[2.19,2.87]	[1.34,2.54]	[1.70,2.51]	[1.45,2.53]	[2.18,3.13]
**Education (None)**						
Primary	0.97	0.98	1.04	1.07	0.86*	0.97
	[0.88,1.08]	[0.92,1.03]	[0.90,1.20]	[0.99,1.16]	[0.76,0.98]	[0.90,1.05]
Secondary	1.02	0.93*	1.17*	1.08*	1.12*	1.08*
	[0.93,1.11]	[0.89,0.98]	[1.04,1.32]	[,1.16]	[1.01,1.24]	[1.01,1.15]
Higher	1.00	0.88**	1.31***	1.09***	1.29***	1.24***
	[0.89,1.12]	[0.80,0.97]	[1.13,1.53]	[1.04,1.14]	[1.13,1.48]	[1.11,1.39]
**Occupation (None)**						
Blue Collar	1.08	1.03	1.02	0.98	1.08	1.14***
	[,1.16]	[0.97,1.10]	[0.93,1.13]	[0.90,1.07]	[0.99,1.18]	[1.06,1.23]
White Collar	1.11*	1.19***	1.12**	1.17***	1.05	1.11***
	[1.01,1.22]	[1.14,1.25]	[1.04,1.21]	[1.10,1.24]	[0.93,1.18]	[1.05,1.17]
**Has insurance (No)**						
Yes	1.18***	1.15***	1.33***	1.19***	1.13**	1.13***
	[1.10,1.26]	[1.09,1.20]	[1.22,1.45]	[1.11,1.26]	[1.04,1.22]	[1.07,1.20]
**Radio use (Not at all)**						
Less Than Once /Week	0.99	1.05	1.09	1.16**	1.17*	1.41***
	[0.87,1.11]	[0.97,1.13]	[0.93,1.27]	[1.04,1.29]	[1.02,1.35]	[1.29,1.54]
At Least Once /Week	1.09	1.05	1.16*	1.24***	1.28***	1.42***
	[0.98,1.21]	[0.97,1.14]	[1.01,1.33]	[1.12,1.38]	[1.13,1.44]	[1.29,1.55]
Almost Every Day	1.20**	1.32***	1.45***	1.36***	1.76***	1.70***
	[1.07,1.35]	[1.21,1.45]	[1.26,1.67]	[1.21,1.53]	[1.55,1.98]	[1.53,1.89]
**TV use (Not at all)**						
Less Than Once/ Week	1.03	1.27***	0.98	1.23***	1.18	1.18***
	[0.88,1.22]	[1.18,1.37]	[0.77,1.23]	[1.10,1.36]	[0.96,1.44]	[1.07,1.30]
At Least Once/Week	1.14	1.27***	1.08	1.15**	1.19*	1.23***
	[,1.30]	[1.19,1.36]	[0.90,1.31]	[1.05,1.27]	[1.01,1.41]	[1.13,1.33]
Almost Every Day	1.16**	1.42***	1.18*	1.40***	1.25**	1.27***
	[1.04,1.30]	[1.34,1.50]	[1.01,1.38]	[1.29,1.51]	[1.09,1.44]	[1.18,1.37]
**Religion (Hindu)**						
Muslim	1.30***	1.24***	1.26***	1.26***	1.51***	2.10***
	[1.21,1.39]	[1.17,1.31]	[1.15,1.39]	[1.16,1.36]	[1.39,1.64]	[1.97,2.25]
Other	1.19***	0.90***	1.14*	0.98	1.59***	1.28***
	[1.09,1.30]	[0.85,0.96]	[1.02,1.28]	[0.91,1.06]	[1.44,1.75]	[1.20,1.37]
**Wealth quintile (Poorest)**						
Poorer	1.18	1.30***	1.08	1.20***	1.09	1.47***
	[0.96,1.44]	[1.23,1.38]	[0.81,1.45]	[1.10,1.31]	[0.83,1.44]	[1.35,1.60]
Middle	1.28*	1.64***	1.20	1.60***	1.44**	2.12***
	[1.06,1.55]	[1.54,1.75]	[0.91,1.59]	[1.46,1.76]	[1.11,1.87]	[1.94,2.32]
Richer	1.52***	2.00***	1.39*	1.92***	1.68***	2.72***
	[1.26,1.83]	[1.86,2.15]	[1.06,1.84]	[1.73,2.13]	[1.30,2.16]	[2.47,2.99]
Richest	1.89***	2.83***	1.62***	2.59***	2.02***	4.29***
	[1.56,2.29]	[2.61,3.08]	[1.22,2.14]	[2.31,2.91]	[1.56,2.61]	[3.86,4.77]
**Household head’s sex (Male)**						
Female	1.05	1.04	1.10	1.10*	1.11*	1.04
	[0.96,1.14]	[0.99,1.11]	[0.99,1.23]	[1.02,1.19]	[1.01,1.22]	[0.97,1.12]
**Healthcare decision maker (Respondent)** Alone						
Respondent & Husband/Partner	1.09	1.12*	1.13*	1.13**	0.94	0.92*
	[1.00,1.18]	[1.03,1.23]	[1.01,1.27]	[1.04,1.23]	[0.86,1.04]	[0.86,0.99]
Husband/Partner Alone	1.10	0.99	1.16*	1.15**	0.91	0.84***
	[0.99,1.21]	[0.92,1.06]	[1.02,1.33]	[1.05,1.27]	[0.82,1.02]	[0.77,0.91]
**Husband’s Education (None)**						
Primary	1.07	1.03	1.13	1.01	1.04	0.93
	[0.95,1.20]	[0.97,1.10]	[0.96,1.32]	[0.95,1.07]	[0.90,1.21]	[0.86,1.01]
Secondary	1.14*	1.07*	1.07	1.04	1.14*	0.98
	[1.03,1.26]	[1.02,1.13]	[0.93,1.24]	[0.96,1.12]	[1.00,1.29]	[0.91,1.05]
Higher	1.06	0.90*	1.00	0.90	1.11	0.86**
	[0.94,1.20]	[0.83,0.99]	[0.84,1.18]	[0.80,1.01]	[0.96,1.29]	[0.78,0.96]

Exponentiated coefficients; 95% confidence intervals in [] brackets. Reference categories in () brackets.

* *p* < 0.05,

** *p* < 0.01,

*** *p* < 0.001

Given the well-established evidence on urban-rural difference in healthcare seeking behavior [31, 32], the multivariable models were further stratified by residence. Current findings show that the strength of the associations varied considerably between urban and rural population for most of the variables. For instance, the association between age and cervix, breast and oral cavity screening were noticeably higher among rural residence across all age groups. Urban women in the ‘other’ religion category had higher odds [Odds ratio = 1.19, 95%CI = 1.09,1.30] of having cervix test while the odds were lower [Odds ratio = 0.90, 95%CI = 0.85,0.96] for rural residents. This observation indicates that urban and rural population different degrees of sensitivity to the social determinants of health factors, and should therefore be analyzed separately.

**Fig 4:**
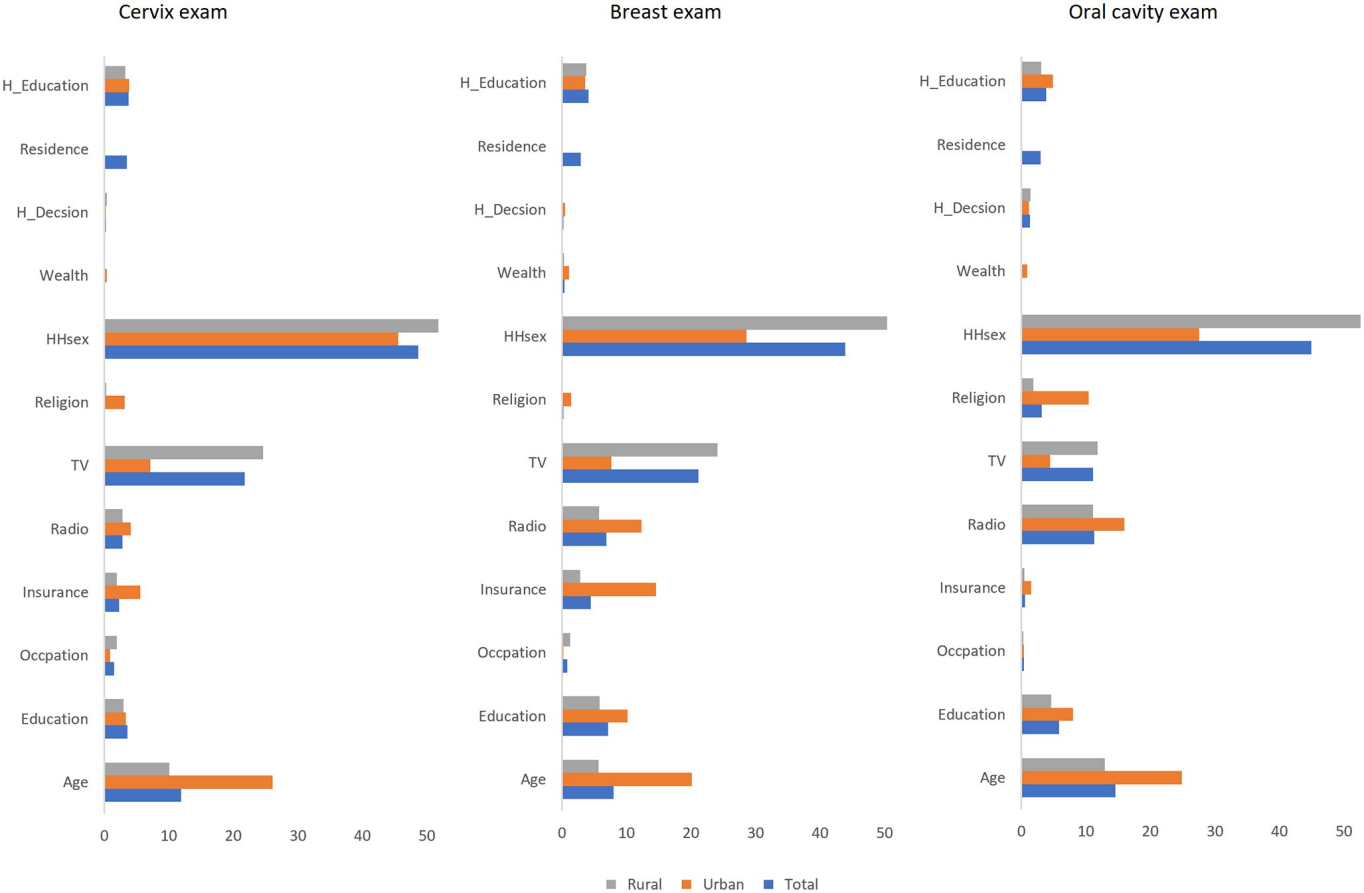
Percent contribution of the explanatory variables to the outcomes.

Fig 4 shows the percent contribution of all the explanatory variables to the outcome variables. It is evident that household head’s sex had the highest contribution to the variability of all three types of cancer screening. The relative percentage varied for urban and rural residents, especially for TV use, health insurance ownership and age. Specifically, the relative contribution of TV use was noticeably higher among rural residents, where that of radio and health insurance ownership was higher among urban residents. Although, the media channels might be communicating the same messages, the urban-rural variance might be explained by differences in people’s choice for programs and their way of interpretation and understanding of those messages.

## Discussion

The present study aimed to measure the prevalence and socioeconomic correlates of cervical, breast and oral cancer screening uptake in India based on a nationally representative sampling from National Family Health Survey. We found that the prevalence of cervical cancer screening was highest among the three, with more than one-fifth reporting having the test, followed by oral and breast cancers. We also observed that less than a fifth of the women had at least one test and 3.3% had all three screening. In comparison with previous studies, the percentage of cervical cancer screening in our study was higher than that found in Kenya (16.4%) [26], Uganda (4.8%) [25]. The finding of breast cancer screening rate being the lowest despite being relatively easier to diagnose and the most common form of cancer among women in many parts in the country [33]. Early detection through uptake of screening services is the key strategy to control the burden of breast cancer attributable to morbidity and mortality. Nonetheless, there is no definitive protocol for mammography for primary care practitioners to follow in the country so far [33].

Apart from the lack of essential healthcare infrastructure, such as diagnostic devices and skilled professionals, the uptake of screening services can also be constrained by socioeconomic factors. Findings from existing studies show that these factors can include age, sex, education, financial situation. Socioeconomic disparities in healthcare service use is a common issue in India, and especially for health services that are considered critical and require a high level of compliance for the preventive (such as screening) measurements to succeed. In population-based studies, these factors are generally classified into enabling and predisposing factors. From our findings, a number of sociodemographic factors were found to be associated with the uptake of cervical, breast and oral cavity cancer screening services including higher age groups, higher education, having white-collar occupation, health insurance, household wealth quintile, exposure to mass media channels, household head’s sex, and place of residence. These findings altogether reflect the protective influence of better socioeconomic standing on the uptake of cancer screening services.

Women with higher education and engagement professional occupations are more likely to be aware of their health conditions as well as the necessity of adopting preventive measures. So far, there is not sufficient research evidence on the barriers to cancer screening uptake among Indian women [34–36]. Evidence from the available small-scale studies reveals that there is a lack of awareness regarding the services and the risk factors of cancer among women. A community based, cross-sectional study carried out in a resettlement colony in South Delhi reported that only 53 percent of the women were aware that breast cancer could be detected early, and only 35 percent were aware of the risk factors. The study concluded that awareness about breast cancer is low, and there is a need for awareness generation programs to educate women about breast cancer to promote early detection [37].

Given the role of health awareness in the uptake of screening services [38, 39], we included three mass media channels including radio and TV in the analysis based on the prior researches that found a significant link between media access and reproductive and other healthcare services utilization [40, 41].

In LMICs, where mobile phone and internet penetration is comparatively low, exposure to traditional mass media functions as a critical source of health communication. We found that compared with women who reported not listening to radio at all, those who reported listening at least once and more than once per week had significantly higher odds of receiving screening services of all three types. Similarly, positive associations were observed for TV viewing as well. From these findings, it is suggestible that health knowledge enhancement programs through traditional media channels can be beneficial in improving the uptake of cancer screening services. The possible explanation behind this could be that women who learn about the risk factors and preventive services about cancer are more likely to discuss it with others and take precautionary steps. Social media communication is increasingly becoming a useful tool for improving health awareness among the general population [42, 43] and can prove to be useful in the context of cancer screening services as well.

Of note, we observed a strong influence of household head’s sex in the uptake of all three types of screening services. As shown in Table 3, the odds of having all three types of screening were higher among women-headed households. This is perhaps due to the fact that female-headed households are more likely to be able to recognize reproductive and other health issues unique to women. Better recognition of the potential health issues is a key driving factor in adopting preventive health behavior. Although the current analysis is unable to explain whether or why the male-headed households are more or less likely to be able to take preventive measures of women’s health issues, it is however assumable that in a low-resource setting with low literacy rates, men and women are not as likely to as be aware of each other’s health issues as it’d be expectable in a developed country setting where the average health literacy is usually higher among the general population. Another possible mechanism could be that women who themselves are households’ heads are more likely to have control over healthcare decision-making as well as affordability of care [44]. Regardless of the potential mechanisms, this finding indicates that men and women in male-headed households might need to be made more aware of women’s cancer screening needs.

To our knowledge, this study is the first to assess the socioeconomic factors associated with cervical, breast and oral cancer screening in a nationally-representative women population in India. Use of stratified analyses (such as urban-rural) allowed us to have a clearer picture of the associations. Sample size was representative for women of 15–49 years, and therefore the findings are generalizable for women of this age range. This study has several limitations to report as well. First, the data were cross-sectional data which prevents from making any causal inference between the explanatory and outcome measures. Secondly, the data were secondary, which meant that we were not able to select variables for analysis that are not available on the datasets. For instance, women’s knowledge and attitude regarding preventive services could have produced a better picture of the associations. It is important to note that there was no information regarding the frequency and age of the screening services. Also, the data were self-reported and remain subject to recall or reporting bias, also known as social desirability bias. Some participants might have over/underreported their screening uptake status. Despite these limitations, the study provides important insights regarding the prevalence and sociodemographic factors of three common types of cancer screening services which should be of particular interest among researchers and policymakers involved in cancer prevention projects.

## Conclusion

Cancer is a growing public health concern for India especially among women due to lack of awareness and inadequate healthcare infrastructure to provide routine screening services. The present study aimed to measure the prevalence and socioeconomic correlates of cervical, breast and oral cancer screening in Indian women that can be of high interest among practitioners and health policymakers. The findings highlighted important sociodemographic disparities in the uptake of screening behaviour for all three types of cancer. Higher age groups, higher education, having white-collar occupation, health insurance, household wealth quintile, exposure to mass media channels, household head’s sex, and place of residence were found to be significantly associated with the uptake of the screening services. These findings imply a socioeconomic gradient in the uptake of screening services that should be addressed through promoting socially and culturally tailored cancer prevention and awareness programs. Further studies are therefore necessary to generate a fuller picture of the situation including the quality of the health service and factors associated with the accessibility of the available services.

## Abbreviations

CAPI: Computer Assisted Personal Interviewing
GATS: Global Adult Tobacco Survey
NFHS: National Family Health Survey
OCC: Oral Cavity Cancer
SLT: Smokeless Types
